# CD4/CD8 ratios as immune architects – building defenses against HIV: A narrative review

**DOI:** 10.1097/MD.0000000000047396

**Published:** 2026-01-30

**Authors:** Emmanuel Ifeanyi Obeagu, Getrude Uzoma Obeagu

**Affiliations:** a Department of Biomedical and Laboratory Science, Africa University, Mutare, Zimbabwe; b School of Nursing Science, Kampala International University, Ishaka, Uganda.

**Keywords:** antiretroviral therapy, CD4, CD8, HIV, immune architecture, immune surveillance, T lymphocytes

## Abstract

The CD4/CD8 ratio is a pivotal metric in assessing the status of the immune system, crucially implicated in human immunodeficiency virus (HIV) infection dynamics. This review elucidates the multifaceted roles of CD4 and CD8 T cells in immune surveillance and response orchestration. We explore the intricate mechanisms governing their equilibrium and the consequential impact on immune homeostasis. Moreover, we dissect how HIV undermines this delicate balance, precipitating a cascade of immune dysregulation culminating in AIDS progression. Insights into the CD4/CD8 ratio’s prognostic value for disease trajectory and treatment efficacy are discussed, highlighting its potential as a pivotal biomarker in clinical management. Additionally, it interrogates emerging therapeutic modalities leveraging the manipulation of CD4/CD8 ratios as a strategic avenue for HIV intervention. Immunomodulatory agents and gene-based approaches aimed at restoring immune balance hold promise in bolstering host defenses against HIV. By harnessing a deeper understanding of the interplay between CD4 and CD8 T cells, novel therapeutic strategies seek to fortify the immune landscape against HIV-induced immunopathogenesis. This review underscores the significance of CD4/CD8 ratios as not only diagnostic indicators but also as potential targets for therapeutic intervention, heralding new horizons in the battle against HIV/AIDS.

## 1. Introduction

Human immunodeficiency virus (HIV) infection remains a significant global health challenge, with approximately 38 million people living with HIV worldwide according to UNAIDS. Despite remarkable advancements in antiretroviral therapy (ART) that have transformed HIV infection into a manageable chronic condition, challenges persist in achieving viral suppression and immune reconstitution. Central to the pathogenesis of HIV infection is the progressive depletion of CD4-positive T cells, leading to immunodeficiency and susceptibility to opportunistic infections. However, recent research has underscored the importance of not only CD4 T cells but also CD8 T cells in orchestrating immune responses and maintaining immune homeostasis. The CD4/CD8 ratio, defined as the ratio of CD4-positive to CD8-positive T lymphocytes, serves as a critical indicator of immune system health and function. Alterations in this ratio have been associated with various disease states, including HIV infection, where it reflects the balance between viral replication and immune control. While a low CD4/CD8 ratio is a hallmark of HIV-induced immune suppression, the dynamics of CD4 and CD8 T cells in response to HIV infection are complex and multifaceted. Understanding the nuanced interplay between CD4 and CD8 T cells is essential for unraveling the immunopathogenesis of HIV infection and identifying novel therapeutic targets.^[1-4]^

### 1.1. Aim

The aim of this review is to comprehensively examine the significance of CD4/CD8 ratios in the context of HIV infection, elucidating their roles in immune function, disease progression, and treatment outcomes.

### 1.2. Rationale

The rationale for this review stems from the critical importance of understanding the CD4/CD8 ratio dynamics in the context of HIV infection. Despite advancements in HIV treatment, immune dysregulation remains a significant challenge, often leading to disease progression and complications. By elucidating the roles of CD4 and CD8 T cells in immune function and their intricate balance, we can gain insights into the mechanisms driving HIV pathogenesis and identify potential targets for therapeutic intervention. Furthermore, exploring the prognostic value of CD4/CD8 ratios in predicting disease progression and treatment outcomes can inform clinical decision-making and optimize patient care. Additionally, as novel therapeutic approaches aimed at modulating CD4/CD8 ratios continue to emerge, a comprehensive understanding of their underlying mechanisms and potential efficacy is essential for advancing HIV management strategies. Thus, this review aims to consolidate current knowledge and highlight future directions in leveraging CD4/CD8 ratios as immune architects in the fight against HIV/AIDS.

## 2. Review methodology

### 2.1. Search strategy

A comprehensive search strategy was developed to identify relevant literature from electronic databases such as PubMed, MEDLINE, Embase, and Web of Science. Search terms included combinations of “CD4/CD8 ratio,” “HIV,” “immune function,” “disease progression,” “prognostic marker,” and “therapeutic intervention.” Boolean operators (AND, OR) were utilized to refine searches, and filters were applied to restrict results to peer-reviewed articles published in English.

### 2.2. Selection criteria

Articles were screened based on predefined inclusion and exclusion criteria. Inclusion criteria encompassed studies investigating CD4/CD8 ratios in HIV-infected individuals, their roles in immune modulation, disease progression, and therapeutic interventions. Exclusion criteria included studies unrelated to HIV infection, non-peer-reviewed sources, and articles not available in full-text.

### 2.3. Data extraction

Data extraction was performed independently by 2 reviewers using a standardized form. Extracted data included study characteristics (e.g., author, year of publication), participant demographics, CD4/CD8 ratio measurements, immune outcomes, disease progression markers, and therapeutic interventions. Discrepancies were resolved through consensus or consultation with a third reviewer.

### 2.4. Quality assessment

The quality of included studies was assessed using appropriate tools such as the Newcastle-Ottawa Scale for observational studies and the Cochrane Risk of Bias tool for randomized controlled trials. Studies were evaluated based on criteria including study design, sample representativeness, outcome assessment, and statistical analysis.

### 2.5. Data synthesis and analysis

Data synthesis involved summarizing key findings from included studies, identifying patterns, and evaluating the strength of evidence. Quantitative data were synthesized using descriptive statistics, while qualitative data were analyzed thematically. Subgroup analyses and meta-analyses were conducted where feasible, considering heterogeneity between studies.

### 2.6. Ethical approval

Not applicable.

### 2.7. CD4 T cells and CD8 T cells in HIV

CD4-positive T cells, often referred to as “helper” T cells, play a central role in orchestrating immune responses against pathogens, including viruses, bacteria, and fungi. These versatile lymphocytes serve as the linchpin of adaptive immunity, coordinating the activities of various immune cells to mount effective defense mechanisms. The hallmark function of CD4 T cells lies in their ability to recognize antigens presented by antigen-presenting cells (APCs), such as dendritic cells and macrophages, through their T cell receptors in conjunction with major histocompatibility complex class II molecules. Upon antigen recognition, CD4 T cells undergo activation and differentiation into distinct subsets, each specialized in executing specific effector functions tailored to combat different types of pathogens. Th1 cells, characterized by the secretion of interferon-γ, are pivotal in mediating cellular immunity against intracellular pathogens, including viruses and intracellular bacteria. Th2 cells, on the other hand, secrete interleukin-4 (IL-4) and IL-13, driving humoral immunity and facilitating the elimination of extracellular parasites. Additionally, regulatory T cells (Tregs) maintain immune tolerance and prevent autoimmunity by suppressing excessive immune responses. In the context of HIV infection, CD4 T cells are the primary targets of viral infection and depletion, leading to profound immunodeficiency and susceptibility to opportunistic infections. HIV exploits the CD4 molecule as its primary receptor for cellular entry, resulting in the depletion of CD4 T cells through direct viral cytopathic effects and immune-mediated mechanisms. The progressive decline in CD4 T cell numbers compromises immune function and impairs the host’s ability to mount effective immune responses against opportunistic pathogens. Despite the significant depletion of CD4 T cells during HIV infection, a subset of CD4 T cells with regulatory properties, known as Tregs, expands in response to HIV-induced immune activation. Tregs play a dual role in HIV pathogenesis, dampening excessive immune activation and inflammation while also suppressing effective antiviral immunity. The dysregulation of Tregs contributes to immune dysfunction and may impede efforts to control viral replication and restore immune homeostasis.^[5-7]^

CD8-positive T cells, also known as cytotoxic T lymphocytes (CTLs), represent the frontline soldiers of the immune system in the battle against viral infections. These specialized lymphocytes play a pivotal role in directly targeting and eliminating virus-infected cells, thereby curbing viral replication and dissemination within the host. Central to the cytotoxic function of CD8 T cells is their ability to recognize and destroy infected cells through the recognition of viral antigens presented on the surface of infected cells in association with major histocompatibility complex class I (MHC-I) molecules. Upon encountering viral antigens, CD8 T cells undergo activation and differentiation into effector CTLs, armed with cytotoxic machinery including perforin and granzymes. Perforin facilitates the entry of granzymes into target cells, leading to apoptosis and subsequent elimination of infected cells. This mechanism allows CD8 T cells to directly eradicate virus-infected cells, thereby limiting viral spread and containing the infection. In the context of HIV infection, CD8 T cells play a critical role in controlling viral replication and maintaining viral latency. Despite the persistent antigenic stimulation posed by HIV, a subset of HIV-specific CD8 T cells mounts effective immune responses, suppressing viral replication and preventing disease progression in a subset of individuals termed “elite controllers.” These individuals exhibit robust HIV-specific CD8 T cell responses characterized by polyfunctionality and cytotoxic capacity, enabling them to effectively control viral replication in the absence of ART. However, HIV employs various mechanisms to evade CD8 T cell-mediated immune responses, including viral escape mutations, downregulation of MHC-I molecules, and induction of T cell exhaustion. These mechanisms undermine the effectiveness of CD8 T cell responses and contribute to viral persistence and immune evasion. Additionally, chronic immune activation and inflammation during HIV infection impair CD8 T cell function and contribute to immune exhaustion, further compromising antiviral immunity.^[8,9]^

### 2.8. Synergy and interdependence

The immune system operates as a complex network of interconnected cells and molecules, orchestrated by a delicate balance of synergistic interactions and interdependencies. At the heart of this intricate system lies the collaboration between CD4 and CD8 T cells, whose concerted efforts form the foundation of immune architecture and defense against pathogens, including HIV. CD4 T cells, aptly termed “helper” T cells, serve as the architects of immune responses, directing and coordinating the activities of various immune cells through cytokine signaling and cell-cell interactions. By recognizing antigens presented by APCs, CD4 T cells initiate and regulate both cellular and humoral immune responses, thereby orchestrating the multifaceted defense mechanisms required for pathogen clearance. In tandem, CD8 T cells, known as cytotoxic T lymphocytes (CTLs), function as the frontline soldiers in viral combat, directly targeting and eliminating virus-infected cells. Their ability to recognize viral antigens presented on infected cells enables CD8 T cells to deploy cytotoxic mechanisms, culminating in the destruction of infected cells and containment of viral spread within the host. The synergy between CD4 and CD8 T cells extends beyond their individual roles, encompassing a dynamic interplay essential for effective immune function. CD4 T cells provide crucial support to CD8 T cells by orchestrating their activation, differentiation, and migration to sites of infection. Conversely, CD8 T cells reciprocate by enhancing the function and survival of CD4 T cells through feedback mechanisms, thereby reinforcing immune responses and promoting immune homeostasis. In the context of HIV infection, this intricate balance is disrupted as the virus targets and depletes CD4 T cells, undermining immune function and compromising host defenses. The loss of CD4 T cells not only impairs the helper function crucial for CD8 T cell activation but also compromises the overall integrity of the immune system, leading to immunodeficiency and susceptibility to opportunistic infections. Despite these challenges, therapeutic interventions aimed at restoring the synergy between CD4 and CD8 T cells hold promise for combating HIV and restoring immune function. Strategies targeting immune checkpoints, such as PD-1/PD-L1, aim to rejuvenate exhausted T cells, including both CD4 and CD8 T cells, thereby enhancing their antiviral responses. Additionally, approaches aimed at modulating cytokine signaling and promoting T cell survival offer potential avenues for bolstering immune defenses against HIV.^[2,10-13]^

### 2.9. CD4 and CD8 T cells as architects of adaptive immunity

CD4 and CD8 T cells stand as the architects of adaptive immunity, orchestrating a coordinated defense against pathogens and maintaining immune homeostasis. These 2 subsets of T lymphocytes play complementary yet distinct roles in immune surveillance, activation, and regulation, collectively shaping the adaptive immune response to confront diverse challenges, including infections and cancer. CD4 T cells, often referred to as “helper” T cells, serve as master regulators of immune responses. Their primary function lies in recognizing antigens presented by APCs via major histocompatibility complex class II molecules. Upon activation, CD4 T cells undergo differentiation into various effector subsets, including Th1, Th2, Th17, and Tregs, each equipped with specialized functions tailored to combat specific types of pathogens or maintain immune tolerance. Th1 cells, characterized by the secretion of interferon-γ, play a pivotal role in mediating cellular immunity against intracellular pathogens, such as viruses and intracellular bacteria. Th2 cells, on the other hand, orchestrate humoral immunity by promoting B cell activation and antibody production, crucial for defense against extracellular parasites. Th17 cells contribute to the host defense against extracellular bacteria and fungi, while Tregs maintain immune tolerance and prevent autoimmune responses by suppressing excessive immune activation. In contrast, CD8 T cells, also known as cytotoxic T lymphocytes (CTLs), specialize in directly targeting and eliminating infected or malignant cells. Their cytotoxic function relies on the recognition of antigenic peptides presented by MHC class I molecules on the surface of target cells. Upon activation, CD8 T cells differentiate into cytotoxic effector cells armed with perforin and granzymes, which induce apoptosis in target cells, effectively clearing the infection or eliminating cancerous cells. The collaboration between CD4 and CD8 T cells is essential for mounting effective immune responses against pathogens. CD4 T cells provide critical signals for the activation and expansion of CD8 T cells, enhancing their cytotoxic activity and promoting memory formation. Conversely, CD8 T cells reciprocate by providing help to CD4 T cells, facilitating their activation and differentiation into effector subsets. In the context of HIV infection, the virus targets and depletes CD4 T cells, leading to immune dysfunction and progression to acquired immunodeficiency syndrome (AIDS). The loss of CD4 T cell help compromises the function of CD8 T cells, impairing their ability to control viral replication and leading to persistent immune activation and inflammation. Efforts to restore immune function in HIV-infected individuals have focused on enhancing the function of both CD4 and CD8 T cells. Immunotherapeutic approaches, including immune checkpoint blockade and adoptive T cell therapy, aim to rejuvenate exhausted T cells and enhance their antiviral activity. Additionally, therapeutic vaccination strategies seek to induce robust CD4 and CD8 T cell responses, capable of controlling viral replication and preventing disease progression.^[14-17]^

### 2.10. Harmony and dissonance in HIV pathogenesis

HIV pathogenesis is a complex interplay between harmony and dissonance within the immune system. While the virus orchestrates a symphony of evasion strategies to persist and replicate, it disrupts the delicate harmony of immune responses, leading to progressive immunodeficiency and disease progression. Understanding these dual dynamics is essential for unraveling the complexities of HIV infection and developing effective therapeutic interventions. At the heart of HIV pathogenesis lies the virus’s ability to exploit the immune system’s regulatory pathways, creating a harmonious environment for viral replication and dissemination. HIV infects CD4-positive T cells, the conductors of immune responses, and hijacks their machinery to facilitate viral replication. As the virus proliferates, it undermines the integrity of the immune system, impairing both innate and adaptive immune responses and fostering a permissive environment for viral persistence. Simultaneously, HIV triggers a cacophony of immune activation and inflammation, fueling a chronic state of immune dissonance. The virus induces aberrant immune activation through various mechanisms, including direct viral cytopathic effects, bystander T cell activation, and microbial translocation from the gut. This persistent immune activation drives the depletion of CD4 T cells, disrupts T cell homeostasis, and promotes the exhaustion of antiviral immune responses, further exacerbating immunodeficiency and disease progression. The dysregulation of immune checkpoints, such as PD-1/PD-L1, represents a key nexus of dissonance in HIV pathogenesis. HIV exploits these inhibitory pathways to evade immune surveillance, leading to T cell exhaustion and functional impairment. The resulting immune dysfunction impairs the ability of the immune system to control viral replication and contributes to persistent viremia and disease progression. Moreover, the discordant interplay between HIV and the host immune system fosters a pro-inflammatory milieu characterized by elevated levels of cytokines, chemokines, and immune activation markers. This chronic inflammation contributes to end-organ damage, immune senescence, and increased risk of non-AIDS-related complications, including cardiovascular disease, neurocognitive impairment, and malignancies. However, amidst the dissonance of HIV pathogenesis, pockets of harmony emerge, exemplified by the phenomenon of elite control. Elite controllers, a rare subset of HIV-infected individuals, maintain undetectable viral loads and stable CD4 T cell counts in the absence of ART. Their ability to control viral replication is attributed to robust and polyfunctional immune responses, including cytotoxic CD8 T cell activity, strong antibody responses, and effective immune regulation.^[18,19]^

The evolving interplay between the virus and the host immune system dictates the trajectory of HIV disease, influencing clinical outcomes and treatment responses. Several key factors contribute to disease progression in HIV, each with profound implications for immune function and overall prognosis. Central to disease progression in HIV is the gradual depletion of CD4-positive T cells, the hallmark of immunodeficiency in HIV/AIDS. As the virus targets and infects CD4 T cells, it undermines immune function, impairing the body’s ability to mount effective immune responses against opportunistic infections and malignancies. The decline in CD4 T cell counts serves as a critical prognostic marker, with lower CD4 counts correlating with increased risk of AIDS-defining illnesses and mortality. Additionally, chronic immune activation and inflammation play a pivotal role in driving disease progression in HIV. Persistent viral replication fuels a state of chronic immune activation, characterized by elevated levels of pro-inflammatory cytokines, chemokines, and immune activation markers. This sustained inflammatory milieu contributes to T cell exhaustion, immune dysfunction, and end-organ damage, accelerating disease progression and increasing the risk of non-AIDS-related complications, including cardiovascular disease, neurocognitive impairment, and metabolic disorders. Furthermore, the emergence of viral escape mutations and the establishment of viral reservoirs pose significant challenges to HIV treatment and disease management. HIV’s high mutation rate and genetic diversity enable the virus to evade immune detection and ART, leading to the emergence of drug-resistant strains and treatment failure. Viral reservoirs, established early in infection, harbor latent virus that persists despite effective ART, serving as a potential source of viral rebound upon treatment interruption and hindering efforts to achieve viral eradication or functional cure. Host factors, including genetic predisposition, immune function, and comorbidities, also influence disease progression in HIV. Host genetic polymorphisms, such as those affecting human leukocyte antigen (HLA) molecules and immune response genes, can impact susceptibility to HIV infection and disease progression. Additionally, coinfections, such as hepatitis C virus and tuberculosis, contribute to accelerated disease progression and increased mortality in HIV-infected individuals, further complicating clinical management and treatment outcomes. Understanding the multifaceted determinants of disease progression in HIV is essential for tailoring therapeutic strategies and optimizing patient care. Early initiation of ART, guided by CD4 T cell counts and viral load measurements, remains the cornerstone of HIV treatment, aiming to suppress viral replication, preserve immune function, and reduce the risk of disease progression and transmission. However, addressing the underlying drivers of immune dysregulation, including chronic inflammation, immune exhaustion, and viral persistence, requires a multifaceted approach, encompassing immune-based therapies, adjunctive treatments, and targeted interventions aimed at restoring immune homeostasis and achieving long-term control of HIV infection. By elucidating the complex interplay between the virus and the host immune system, we can pave the way for innovative therapeutic strategies that mitigate disease progression, improve clinical outcomes, and ultimately achieve durable control of HIV/AIDS.^[19,20]^

Immunological resilience emerges as a critical determinant of long-term outcomes in HIV infection, shaping disease progression, treatment responses, and overall prognosis. Defined as the ability of the immune system to maintain functionality and adaptability in the face of viral challenges, immunological resilience encompasses a spectrum of host factors, immune responses, and therapeutic interventions that collectively influence clinical trajectories and survival outcomes in HIV-infected individuals. A cornerstone of immunological resilience in HIV is the preservation of CD4 T cell function and homeostasis, which serves as a key predictor of long-term clinical outcomes. Individuals with robust CD4 T cell recovery, characterized by sustained increases in CD4 counts following initiation of ART, demonstrate improved immune function, reduced risk of opportunistic infections, and prolonged survival. Conversely, persistent CD4 T cell depletion, despite viral suppression, is associated with increased morbidity, mortality, and progression to AIDS-defining illnesses. Moreover, the durability of viral suppression achieved through ART plays a pivotal role in shaping long-term outcomes in HIV. Early and sustained viral suppression not only prevents disease progression and transmission but also preserves immune function, reduces the risk of treatment failure and drug resistance, and improves overall quality of life. However, interruptions in treatment adherence, emergence of drug-resistant strains, and suboptimal therapeutic regimens can compromise viral suppression, leading to virological rebound, immune decline, and adverse clinical outcomes. In addition to traditional markers of immune function, such as CD4 T cell counts and viral load, emerging biomarkers of immunological resilience offer insights into long-term outcomes and treatment responses in HIV. Biomarkers of immune activation, inflammation, and exhaustion, including soluble markers, cell surface molecules, and gene expression profiles, provide valuable prognostic information and guide therapeutic decision-making, enabling early identification of individuals at risk of disease progression or treatment failure. Furthermore, host factors, including genetic polymorphisms, immune response genes, and comorbidities, contribute to immunological resilience and influence long-term outcomes in HIV. Host genetic variations, such as HLA alleles associated with viral control, cytokine polymorphisms affecting immune function, and immune exhaustion markers, impact susceptibility to infection, disease progression, and response to therapy. Additionally, coinfections, such as hepatitis B and C viruses, tuberculosis, and non-communicable diseases, exacerbate immune dysfunction, accelerate disease progression, and increase the risk of mortality in HIV-infected individuals, highlighting the importance of comprehensive clinical management and integrated care approaches. Therapeutic interventions aimed at enhancing immunological resilience offer promising avenues for improving long-term outcomes in HIV. Immune-based therapies, including therapeutic vaccines, immune checkpoint inhibitors, and immune modulators, aim to restore immune function, mitigate chronic inflammation, and augment antiviral immune responses, thereby promoting durable control of HIV infection and reducing the burden of comorbidities.^[21-24]^

### 2.11. Viral proteins involved in CD4 interaction: gp120 and gp41

In the context of HIV infection, 2 key viral proteins, gp120 and gp41, play crucial roles in the interaction with CD4 receptors on host T cells.

#### 2.11.1. gp120

gp120 is a glycoprotein found on the surface of the HIV envelope.^[25]^ It is approximately 120 kDa in size and is heavily glycosylated. gp120 is responsible for the initial attachment of the virus to the host cell. It interacts specifically with the CD4 receptor on T cells and other immune cells. The binding of gp120 to CD4 induces a conformational change in the gp120 molecule, which exposes additional sites that can bind to chemokine co-receptors, primarily CCR5 or CXCR4.^[26]^ This interaction is essential for the entry of HIV into the host cell. After binding to CD4, gp120’s interaction with co-receptors facilitates the fusion of the viral envelope with the host cell membrane, allowing the virus to enter the cell. Due to its role in the initial stages of HIV infection, gp120 is a major target for vaccine design and therapeutic interventions. Neutralizing antibodies that target gp120 can block its interaction with CD4, potentially preventing HIV entry.^[27]^

#### 2.11.2. gp41

gp41 is a transmembrane glycoprotein that forms a stable association with gp120.^[28]^ It is about 41 kDa in size and is critical for the fusion of the viral and cellular membranes. After gp120 binds to CD4 and co-receptors, gp41 undergoes significant conformational changes that lead to the fusion of the viral envelope with the host cell membrane.^[29]^ This process involves the insertion of gp41 into the host membrane and the formation of a 6-helix bundle that brings the viral and cellular membranes close together. The fusion process mediated by gp41 is a target for several classes of antiviral drugs, such as fusion inhibitors (e.g., Enfuvirtide), which block gp41 from mediating membrane fusion and entry of the virus. gp41 can also modulate immune responses and evade detection by the host immune system, contributing to the virus’s persistence and pathogenesis.

### 2.12. Gene polymorphism in the HLA system and cytokine effects on CD4/CD8 ratios in HIV

#### 2.12.1. Gene polymorphism in the HLA system

The HLA system plays a crucial role in the immune response, particularly in the context of HIV infection.^[30]^ HLA molecules are highly polymorphic, and their variations can significantly influence the immune response to HIV.^[31]^

- HLA class I and II genes:
  ○ HLA class I (e.g., HLA-A, HLA-B, HLA-C): These molecules present endogenous antigens, including viral peptides, to CD8+ T cells. Certain HLA class I alleles are associated with a better control of HIV, resulting in higher CD8+ T cell responses and improved CD4/CD8 ratios.
  ○ HLA class II (e.g., HLA-DR, HLA-DQ, HLA-DP): These molecules present exogenous antigens to CD4+ T cells. Variations in HLA class II genes can influence the activation and differentiation of CD4+ T cells, affecting the overall immune response to HIV.
- Impact on CD4/CD8 ratios:

Individuals with specific HLA alleles (e.g., HLA-B*57, HLA-B*27) have been shown to have better control of HIV replication, leading to preserved CD4+ T cell counts and improved CD4/CD8 ratios. In contrast, certain HLA alleles associated with poor immune responses can lead to accelerated CD4+ T cell depletion and a decreased CD4/CD8 ratio.^[32]^

#### 2.12.2. Cytokine effects on CD4/CD8 ratios

Cytokines are critical mediators of the immune response, and their expression can significantly impact CD4/CD8 ratios during HIV infection.

- Cytokine profiles:
  ○ Pro-inflammatory cytokines: Cytokines such as IL-6, IL-12, and TNF-α can promote CD8+ T cell activation and proliferation. Elevated levels of these cytokines are often observed in HIV-infected individuals and can lead to increased CD8+ T cell counts, which may decrease the CD4/CD8 ratio.^[33]^
  ○ Regulatory cytokines: Cytokines like IL-10 and TGF-β can suppress immune activation and promote the differentiation of Tregs, which can help maintain CD4+ T cell homeostasis. However, high levels of these cytokines can also inhibit effective immune responses against HIV.
- Influence on CD4/CD8 ratios:

A balanced cytokine environment is crucial for maintaining healthy CD4/CD8 ratios. A skewed cytokine profile favoring pro-inflammatory responses may lead to CD4+ T cell depletion and an increased CD8+ T cell response, resulting in a lower CD4/CD8 ratio. In contrast, a cytokine milieu that supports CD4+ T cell survival and function can help maintain or improve CD4/CD8 ratios.

#### 2.12.3. Interactions between HLA polymorphisms and cytokines

The interplay between HLA gene polymorphisms and cytokine production can further influence CD4/CD8 ratios in HIV-infected individuals.^[34]^ Different HLA alleles can affect the expression levels of cytokines, shaping the immune landscape. For instance, individuals with certain HLA-B alleles may produce different levels of cytokines in response to HIV, thereby influencing the activation and differentiation of CD4+ and CD8+ T cells. Understanding the interactions between HLA polymorphisms and cytokine responses can inform strategies to optimize immune responses in HIV-infected individuals. This includes the development of personalized therapies that consider both genetic and immunological factors.

### 2.13. Cytokines and their role in CD4/CD8 balance in HIV infection: focus on IL-7 and IL-15

Cytokines are critical in regulating immune responses, and their levels can significantly impact the CD4/CD8 T cell balance in HIV infection.^[35]^ Among the many cytokines, IL-7 and IL-15 play particularly important roles in maintaining immune homeostasis, promoting CD4+ T cell activation, and influencing the overall progression of HIV disease.

#### 2.13.1. Interleukin-7 (IL-7)

IL-7 is a cytokine crucial for T cell development and homeostasis. It is produced by stromal cells in the bone marrow and thymus, as well as by other cells, such as epithelial cells and dendritic cells. IL-7 is essential for the survival and proliferation of naive and memory T cells, particularly CD4+ T cells. IL-7 promotes the survival of CD4+ T cells by upregulating the anti-apoptotic protein Bcl-2, which prevents programmed cell death. This effect helps maintain a higher CD4+ T cell count in the context of HIV infection.^[36]^ IL-7 stimulates the proliferation of CD4+ T cells through the activation of signaling pathways involving the IL-7 receptor (IL-7R) and common gamma chain (γc). This leads to increased CD4+ T cell activation and expansion, contributing to a healthier CD4/CD8 ratio. Studies have shown that IL-7 therapy can enhance immune reconstitution in HIV-infected individuals, particularly in those with low CD4+ T cell counts. By promoting the expansion of CD4+ T cells, IL-7 can help restore a more favorable CD4/CD8 ratio. Elevated IL-7 levels have been associated with improved CD4 counts and better immune function in HIV-positive patients, indicating its potential as a therapeutic target.

#### 2.13.2. Interleukin-15 (IL-15)

IL-15 is a cytokine that plays a vital role in the activation and proliferation of T cells, particularly CD8+ T cells, and is involved in the generation of memory T cells.^[37]^ It is produced by various cells, including macrophages, dendritic cells, and muscle cells. IL-15 is crucial for the survival and proliferation of CD8+ T cells, supporting their effector functions against viral infections. It enhances the expression of key activation markers and promotes cytotoxic activity. While IL-15 primarily affects CD8+ T cells, it also enhances CD4+ T cell responses by promoting the development of Th1 cells, which produce pro-inflammatory cytokines that can further stimulate immune responses. IL-15 can help preserve CD4+ T cell numbers in the setting of HIV by promoting T cell homeostasis and function. Its role in stimulating CD8+ T cell responses can lead to a reduction in viral load, indirectly benefiting CD4+ T cell counts. Higher IL-15 levels have been associated with better CD4/CD8 ratios and improved overall immune function in HIV-infected individuals. IL-15 therapy has shown promise in enhancing T cell responses and restoring immune balance.^[38]^

#### 2.13.3. Other cytokines influencing CD4/CD8 balance

- IL-2: Stimulates T cell proliferation and enhances CD4+ T cell activation. Its levels can be affected by HIV infection, and supplementation may help restore CD4 counts.^[39]^
- IL-10: An anti-inflammatory cytokine that can inhibit the function of CD4+ T cells and lead to a skewed CD4/CD8 ratio if present in high levels. Balancing IL-10 with pro-inflammatory cytokines is essential for maintaining immune function.
- TNF-α: A pro-inflammatory cytokine that can drive CD8+ T cell expansion but may also contribute to CD4 + T cell depletion in chronic inflammation, affecting the CD4/CD8 ratio.

### 2.14. Chemokines and their effects on CD4/CD8 ratios in HIV

Chemokines are small signaling proteins that play crucial roles in immune cell migration, activation, and homeostasis. They are categorized into 2 main families: CC and CXC chemokines, based on the arrangement of their cysteine residues. In the context of HIV infection, chemokines can significantly influence the CD4/CD8 T cell ratio, affecting immune responses and disease progression.^[40]^

#### 2.14.1. Role of chemokines in HIV pathogenesis

- Chemokine receptors as co-receptors:

CD4 is the primary receptor for HIV entry, but the virus also requires co-receptors, such as CCR5 and CXCR4, which are chemokine receptors. The interaction between HIV and these receptors is critical for viral tropism and pathogenesis. CCR5 is predominantly used by R5-tropic HIV strains, while CXCR4 is used by X4-tropic strains. The expression of these receptors on CD4+ T cells can influence their susceptibility to HIV infection and subsequent immune responses.^[41]^

#### 2.14.2. Chemokines affecting CD4+ T cell dynamics

- CCL3 (MIP-1α), CCL4 (MIP-1β), and CCL5 (RANTES):

These chemokines are known for their ability to attract CD4+ T cells and regulate their activation. They inhibit HIV replication by competing with the virus for binding to CCR5. Higher levels of these chemokines can correlate with better preservation of CD4+ T cells, thereby positively affecting the CD4/CD8 ratio. Studies have shown that individuals with higher levels of CCL3, CCL4, and CCL5 often exhibit better immune control of HIV and more favorable CD4/CD8 ratios.^[42]^

- CCL2 (MCP-1):

CCL2 is implicated in recruiting monocytes and T cells to sites of inflammation. While it can enhance CD4+ T cell recruitment, excessive CCL2 can lead to increased inflammation, potentially contributing to CD4+ T cell depletion. Elevated CCL2 levels in HIV-infected individuals are associated with immune activation and a subsequent decrease in the CD4/CD8 ratio, reflecting chronic inflammation and CD4+ T cell loss.^[43,44]^

#### 2.14.3. Chemokines affecting CD8+ T cell dynamics

- CXCL9 (MIG) and CXCL10 (IP-10):

These CXC chemokines attract activated CD8+ T cells to sites of infection. Increased expression of CXCL9 and CXCL10 is often observed in HIV infection, correlating with enhanced CD8+ T cell activation and proliferation. However, this can also lead to a relative increase in CD8+ T cells compared to CD4+ T cells, potentially lowering the CD4/CD8 ratio. CXCL10 has been associated with viral control and may be indicative of a strong CD8+ T cell response against HIV.

#### 2.14.4. Impact of chemokine imbalance on CD4/CD8 ratios

Chronic HIV infection is characterized by persistent immune activation, which can be driven by the dysregulation of chemokine production. This results in an imbalance between pro-inflammatory and regulatory signals, influencing the CD4/CD8 ratio. Elevated levels of pro-inflammatory chemokines may lead to increased CD8+ T cell activation and proliferation, while simultaneously promoting CD4+ T cell apoptosis, thus contributing to a decreased CD4/CD8 ratio. The interplay between various chemokines is crucial for maintaining immune homeostasis. Disruption of this network, such as through the selective recruitment of CD8+ T cells at the expense of CD4+ T cells, can lead to impaired immune responses and a decrease in the CD4/CD8 ratio.^[44-46]^

Table 1 shows correlation among different cells and molecules in HIV (provided by the authors).

**Table 1:** Correlation among different cells and molecules in HIV.

Cell type/cytokine	Role in HIV infection	Impact on CD4/CD8 ratio	Notes
CD4+ T cells	Primary target for HIV; orchestrate immune responses	Decrease in chronic HIV infection	HIV infection leads to depletion and functional impairment
CD8+ T cells	Cytotoxic response against HIV-infected cells	Increase in chronic infection	Enhanced CD8+ T cell response can lead to reduced CD4/CD8 ratio
IL-17	Promotes survival and proliferation of CD4+ T cells	Increases CD4/CD8 ratio	Enhances immune reconstitution and homeostasis
IL-15	Activates and proliferates CD8+ T cells; supports CD4+ T cell function.	Can stabilize CD4/CD8 ratio	Promotes effector functions; elevated in chronic infection
CCL3 (MIP-1α)	Attracts CD4+ T cells; inhibits HIV entry	Increases CD4/CD8 ratio	Higher levels associated with better immune control
CCL4 (MIP-1β)	Similar role as CCL3; inhibits HIV replication	Increases CD4/CD8 ratio	Correlates with CD4+ T cell preservation
CCL5 (RANTES)	Attracts CD4+ T cells; inhibits CCR5 usage by HIV	Increases CD4/CD8 ratio	Linked to protective immune responses
CCL2 (MCP-1)	Recruits monocytes and T cells; promotes inflammation	Decreases CD4/CD8 ratio	Chronic inflammation can lead to CD4+ T cell depletion
CXCL9 (MIG)	Attracts activated CD8+ T cells to sites of infection	Can decrease CD4/CD8 ratio	High levels may lead to increased CD8+ T cell responses
CXCL10 (IP-10)	Stimulates CD8+ T cell migration; associated with inflammation	Can decrease CD4/CD8 ratio	Indicative of immune activation and viral control
TNF-α	Pro-inflammatory cytokine; can induce CD4+ T cell apoptosis	Decreases CD4/CD8 ratio	Chronic elevation linked to immune activation and dysfunction
IL-10	Anti-inflammatory cytokine; inhibits CD4+ T cell activation	Decreases CD4/CD8 ratio	High levels can lead to immune evasion and imbalance

HIV = human immunodeficiency virus, IL = interleukin.

## 3. Conclusion

HIV infection represents a dynamic interplay between viral replication and host immune responses, shaping the trajectory of disease progression and long-term outcomes. While the virus orchestrates mechanisms to evade immune detection and foster viral persistence, it simultaneously triggers chronic immune activation, inflammation, and immune dysfunction, driving progressive immunodeficiency and disease progression. The loss of CD4-positive T cells, chronic immune activation, and the establishment of viral reservoirs contribute to the gradual deterioration of immune function and increased susceptibility to opportunistic infections and non-AIDS-related complications. However, amidst the challenges posed by HIV infection, pockets of immunological resilience emerge, exemplified by elite controllers and individuals with robust immune responses capable of controlling viral replication.

## Author contributions

**Conceptualization:** Emmanuel Ifeanyi Obeagu.

**Methodology:** Emmanuel Ifeanyi Obeagu, Getrude Uzoma Obeagu.

**Supervision:** Emmanuel Ifeanyi Obeagu.

**Validation:** Emmanuel Ifeanyi Obeagu.

**Visualization:** Emmanuel Ifeanyi Obeagu.

**Writing** – **original draft:** Emmanuel Ifeanyi Obeagu, Getrude Uzoma Obeagu.

**Writing** – **review & editing:** Emmanuel Ifeanyi Obeagu, Getrude Uzoma Obeagu.
